# Study of rapid reorganization of visual neurofunctions with the resting‐state functional MRI in pituitary adenoma patients with vision improvement after transsphenoidal surgery

**DOI:** 10.1002/brb3.1917

**Published:** 2021-01-27

**Authors:** Fuyu Wang, Peng Wang, Yuyang Liu, Tao Zhou, Xianghui Meng, Jinli Jiang

**Affiliations:** ^1^ Department of Neurosurgery Chinese PLA General Hospital Beijing China

**Keywords:** action observation network, default mode network, multisensory system, pituitary adenoma, resting‐state functional magnetic resonance imaging, visual improvement

## Abstract

**Introduction:**

To investigate changes of vision‐related resting‐state activity in pituitary adenoma (PA) patients with visual improvement after transsphenoidal surgery.

**Methods:**

14 PA patients with visual improvement after surgery were enrolled. The resting‐state functional MRI and neuro‐ophthalmologic evaluation were performed before and after the operation. The functional connectivity (FC) of 8 seeds (the primary visual cortex (V1), the secondary visual cortex (V2), the middle temporal visual cortex (MT+), and fusiform gyrus(FG)) was evaluated. A paired *t* test was conducted to identify the differences between the two groups.

**Results:**

Compared with the preoperation counterparts, the PA patients with improved vision exhibited decreased FC with the V1, V2, MT+, FG in the left paracentral lobule, bilateral lingual gyrus, precentral gyrus(BA 4), right superior temporal gyrus(BA 22), left fusiform gyrus, bilateral middle occipital gyrus (BA 19), left cuneus, right inferior occipital gyrus, left superior frontal gyrus, right cuneus, left superior parietal lobule(BA 7),the medulla, right postcentral gyrus, and increased FC in the right middle frontal gyrus, left inferior parietal lobule (BA 40), left declive, right lentiform nucleus, inferior frontal gyrus, right superior frontal gyrus(BA 11), cingulate gyrus(BA 32), right putamen, right thalamus, left medial frontal gyrus, left claustrum, left superior frontal Medial, right rectal gyrus(BA 25) and right parahippocampal gyrus.

**Conclusions:**

The results show most subareas within the visual cortex exhibit decreased functional connectivity. The functional changes in subareas within default mode network (DMN), action observation network (AON) and the multisensory system in PAs propose that vision improvement may lead to function remodeling in higher‐order cortex.

## Graphical abstract

Our results may propose that the functional connectivity decreased in both the dorsal and ventral visual stream after visual restoration in pituitary adenoma patients. The functional changes in subareas within default mode network, action observation network, and the multisensory system in pituitary adenoma patients propose that vision improvement may lead to function remodeling in higher‐order cortex.

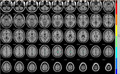

## 1 INTRODUCTION

Neuroplasticity means the inherently dynamic biological ability of the central nervous system to develop maturation, to reshape structurally and functionally in related to experience, and to adapt after injury. The human visual cortex is the good tool to study the development and plasticity of the neocortex. The experience‐dependent neural plasticity is mainly present in the developing visual cortex. The human visual cortex demonstrates immature at birth, develops the deep perception at about 6 months after birth, and acquires maturation in late childhood (Kovács, 2000). However, the potential plasticity is presented in the adult visual cortex (Chen et al., 2010). Therapeutic interventions can also trigger plastic changes in the aging visual cortex by restoring vision (Lou et al., 2013).

The visual cortex comprises a primary visual field (V1) and numerous extrastriate/association visual areas (Felleman & Van Essen, 1991). The innervation of the V1 by the thalamus is the lateral geniculate nucleus (LGN), and the middle temporal cortex (MT+) is the inferior pulvinar (PIm) and LGN [Nassi & Callaway, 2006; Nassi et al., 2006]. These retinothalamic pathways to area MT + could be important drivers/modulators of visual perception in the adult (Laycock et al., 2007).

The two‐stream hypothesis is an influential and widely accepted model of visual information processing (Ungerleider & Mishkin, 1982). From V1, the dorsal stream (the “how pathway”) extends to area MT+ (V5), and on to the posterior parietal cortex, in which way spatial/motion vision was processed. The ventral pathway (the “what pathway”) continued to V4 and terminated in the inferior temporal cortex, in which way form/object vision was processed. The ventral stream pathway included areas with strong preference for faces in the fusiform face area and occipital face area, for body parts in the extrastriate body area. Many of the areas between two parallel streams are very likely interconnected (Braddick et al., 2000; Farivar et al., 2009).

“Top‐down” refers to cognitive influences and higher‐order representations that impinge upon earlier steps in information processing. The top‐down signal can facilitate the integration of objects and backgrounds in the visual scene to make a stable representation of the objects within it (Gilbert & Li, 2007).

FMRI is an indirect measure of neuronal function by measuring localized changes in the oxygenation of blood hemoglobin. It is noninvasive and well tolerated by patients. Resting‐state functional magnetic resonance imaging (RS‐fMRI) is used to evaluate functional connectivity (FC) between brain regions when patients are at rest (Fox & Raichle, 2007; Pillemer et al., 2017). FC refers to the temporal correlation between spatially remote neurophysiological events (Friston et al., 1993). FC analyses based on BOLD signal are particularly promising because they can offer high spatial resolution and high spatial specificity relative to where the corresponding changes in neurophysiological signals take place compared with all other noninvasive imaging modalities (Shmuel et al., 2007). FC analyses can potentially further understanding of neuroanatomical models (Fox & Raichle, 2007).

Pituitary adenoma (PA) may compress the optic chiasm, optic nerve, or optic tract. The patients with PA often present with impaired vision. The transsphenoidal surgery is minimally invasive and does no harm to the visual cortex and other closed region. After the removal of tumor, many patients’ vision was improved. In those PA patients who had improved vision at about 3 months after the operation, the results showed that regional homogeneity (ReHo) decreased or increased within the visual cortex and some brain region (Qian et al., 2015). In clinic, some patients can acquire improved vision right away after surgery. During the early time after surgery, however, the change of neurofunction in visual cortex and higher cognitive cortex is still yet to be explored so far. In literature, only one study presented the neurofunction change of vision areas after visual restoration after 3 days following surgery (Giulia et al., 2012). But in this research, only one patient was recruited, and the task‐fMRI was used to evaluate the brain function. So we recruited more PA patients with improved vision at about 3 days after transsphenoidal operation. We used a priori defined regions of interest (ROIs) in areas V1, V2, MT+, and FG (fusiform gyri) to analyze RS‐fMRI data. Our study is to evaluate the changes in the vision‐related resting‐state network in PA patients. Furthermore, we plan to explore the plasticity of some specific subareas within the visual cortex and higher cognitive networks after restoration of vision.

## 2 MATERIALS AND METHODS

### 2.1 Subjects

16 patients with pituitary adenoma were enrolled in this study. All those patients with the visual damage underwent transsphenoidal tumor resection surgery and got restored vision right away after the surgery. PA patients were recruited according to the inclusion criteria: Age ranged from 18–65 years; the corrected vision acuity was below 1.0 (20/20) before the operation, ophthalmologic diseases or other intracranial lesions that affected the visual pathway or cortex were ruled out; vision improvement at the 3 day after operation (the corrected vision acuity improved by more than 0.2 at least unilaterally) was required; and no severe electrolyte disturbance, hypopituitarism or other complications presented after the operation. This study was approved by the Ethics Committee of Hospital. Written informed consent was obtained from the patients.

### 2.2 Data acquisition

Images were acquired one day preoperatively and three days postoperatively on a 1.5 T MR system (Espree, Siemens Medical Solution, Erlangen, Germany) in the diagnostic room of iMRI brain suite, which was described in detail previously(Chen et al., 2012). Foam pad was used to minimize head movement, and earplugs were set to decrease acoustic noise during scanning. During the RS‐fMRI scan, the patients were instructed to remain motionless and keep their eyes closed and not to think systematically. RS‐fMRI data were acquired using an echo‐planar image pulse sequence (slice thickness = 4.5 mm, flip angle = 90°, and field of view [FOV] =224 mm × 224 mm, TR: 2,000 ms TE: 45 ms.)

### 2.3 Clinical and neuro‐ophthalmologic assessments

We evaluated the cognition of all patients using the mini‐mental state examination prior to the operation. The patients underwent neuro‐ophthalmologic examination within 2 days prior to the operation and at approximately 3 days after the operation. We measured the best‐corrected visual acuity for distance with the E chart and made report in the decimal scale. We performed the ophthalmic fundus examination with a nonmydriatic retinal camera (Topcon, Japan).

### 2.4 RS‐fMRI analysis

#### 2.4.1 Data preprocessing

We preprocessed the RS‐fMRI data using SPM8 (http://www.fil.ion.ucl.ac.uk/spm) and a pipeline analysis toolbox, DPARSF (http://www.restfmri.net/) (Yan & Zang, 2010). The first ten volumes were discarded for signal equilibrium before the longitudinal magnetization reached a steady state. The next data analysis procedures included slice timing correction, head motion correction, normalization, smoothing, removing linear tread, and filtering (0.01–0.08 Hz).

#### 2.4.2 Analysis of FC

ROIs were taken from the literature (Amunts et al., 2000; Caspers et al., 2013; Kolster et al., 2010), and they were defined as 6‐mm radius spheres in both hemispheres. We included 8 seeds to assess FC (Table 1). These seeds were selected within the occipital cortex (V1, V2, MT+, and FG).

**TABLE 1:** ROIs

	Left hemisphere			Right hemisphere			Literature reference
	X	Y	Z	X	Y	Z	
Visual areas							
V1	−10	−77	3	20	−73	2	Amunts et al. (2000)
V2	−13	−75	6	23	−71	6	Amunts et al. (2000)
MT(V5)	−48	−75	8	46	−78	6	Kolster et al. (2010)
FG	−30	−76	−9	33	−73	11	Caspers et al. (2013)

#### 2.4.3 FC and statistical analysis

First, noise‐related variance, including six head motion parameters, the global mean signal, the white matter signal, and the cerebral spinal fluid (CSF) signal, was removed from the preprocessed data by linear regression analysis. The images were then spatially smoothed with a 6‐mm FWHM Gaussian kernel. The individual FC maps were transformed to z‐maps to improve data normality. A paired *t* test was performed on the z‐maps to show significant differences in correlation between the two groups. The AlphaSim method was selected to correct for multiple comparisons. The corrected value of *p* < .05 (uncorrected *p* < .001 and a minimum of 40 voxels in a cluster) was used as the threshold.

## 3 RESULTS

### 3.1 Studied population

According to the inclusion criterion, 16 patients were recruited in our study. As a result of head motion or the lack of sufficient data after scrubbing, 2 patients were excluded; thus, 14 patients (male/female 7:7) were included in the final analyses. Mean age was 46.3 (range 24–62 years). The main demographic and clinical characteristics of patients are listed in Table 2.

**TABLE 2:** The main demographic and clinical characteristics of the patients

No.	Gender	Age (years)	Vision impairment duration and side	Visual acuity			
				Preoperative		Postoperative	
				Left	Right	Left	Right
1	Female	44	6 months/right	1	0.2	1	0.5
2	Female	54	2 months/bilateral	0.15	0.1	0.2	0.3
3	Female	49	10 months/left	0.5	1	0.8	1
4	Male	55	24 months/bilateral	0.4	0.6	0.6	0.8
5	Female	46	12 months/bilateral	0.2	0.3	0.5	0.4
6	MALE	65	1 month/right	1	0.6	1	0.8
7	MALE	31	2 months/right	0.8	0.4	0.8	0.6
8	MALE	54	6 months/left	0.6	0.8	0.9	0.8
9	MALE	46	12 months/bilateral	0.3	0.4	0.5	0.5
10	Female	62	12 months/left	0.15	0.8	0.4	0.8
11	Male	52	39 months/left	0.15	0.8	0.4	0.8
12	Male	40	24 months/bilateral	0.5	0.3	0.6	0.5
13	Female	26	0.25 months/bilateral	0.8	0.15	0.8	0.4
14	Female	24	24 months/right	1	0.6	1	0.9

### 3.2 Ophthalmologic evaluation

Detailed results of the ophthalmologic evaluation are reported in Table 2.

### 3.3 RS‐fMRI analysis

#### 3.3.1 Decreased FC in the patients after operation

**FIGURE 1:**
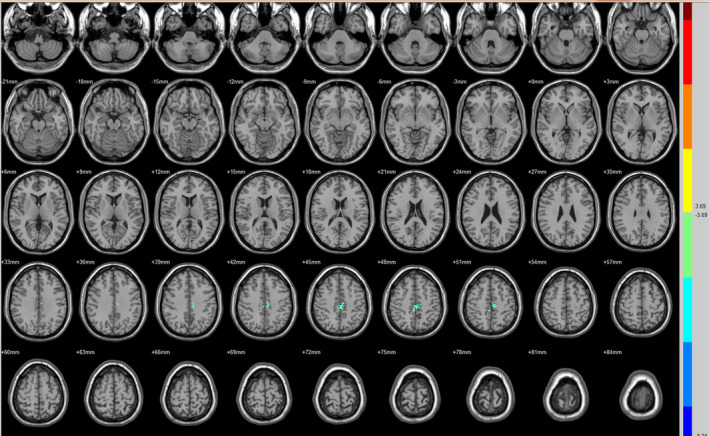
Brain areas exhibited significantly different FCs with the left V1 in PAs (postoperative vs. preoperative)

**FIGURE 2:**
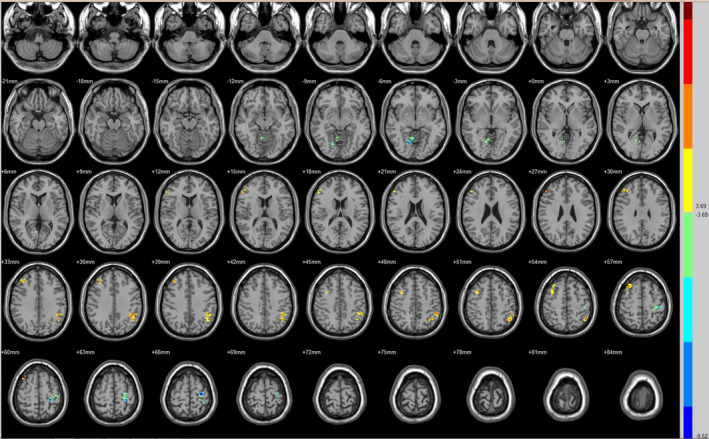
Brain areas exhibited significantly different FCs with the right V1 in PAs (postoperative vs. preoperative)

**FIGURE 3:**
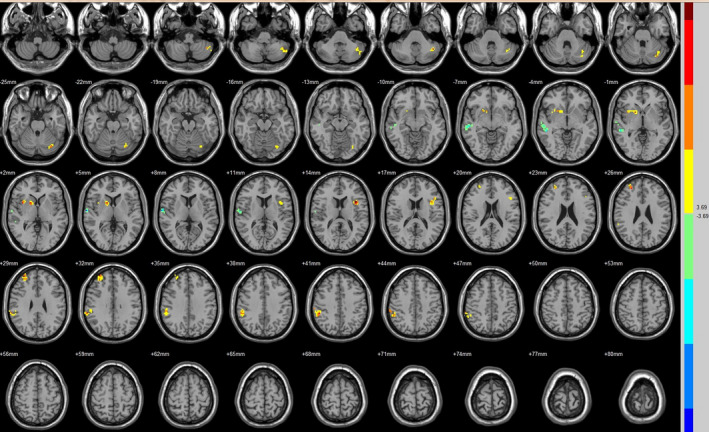
Brain areas exhibited significantly different FCs with the left V2 in PAs (postoperative vs. preoperative)

**FIGURE 4:**
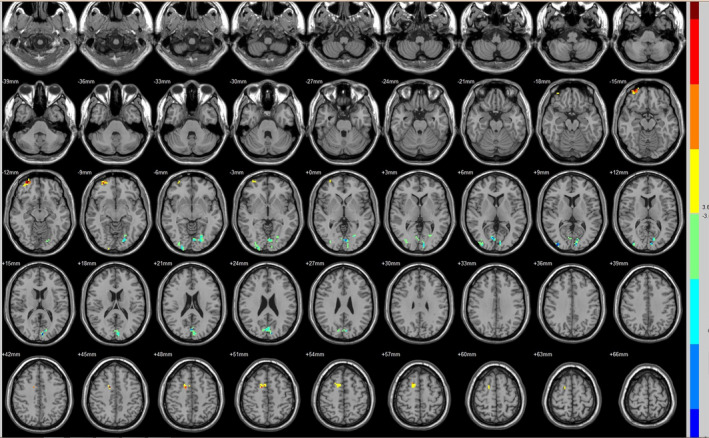
Brain areas exhibited significantly different FCs with the right V2 in PAs (postoperative vs. preoperative)

**FIGURE 5:**
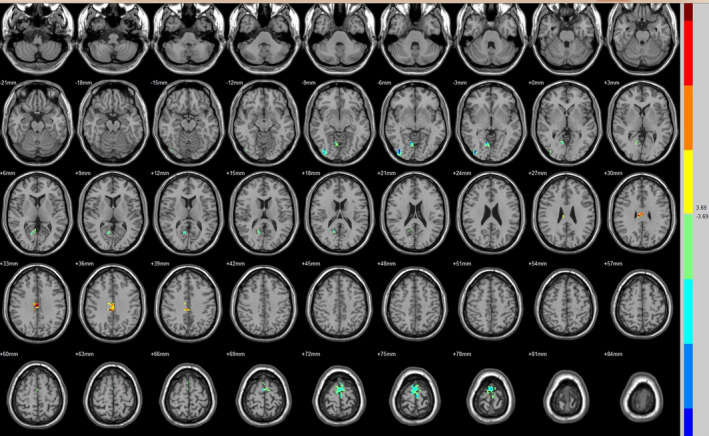
Brain areas exhibited significantly different FCs with the left MT + in PAs (postoperative vs. preoperative)

**FIGURE 6:**
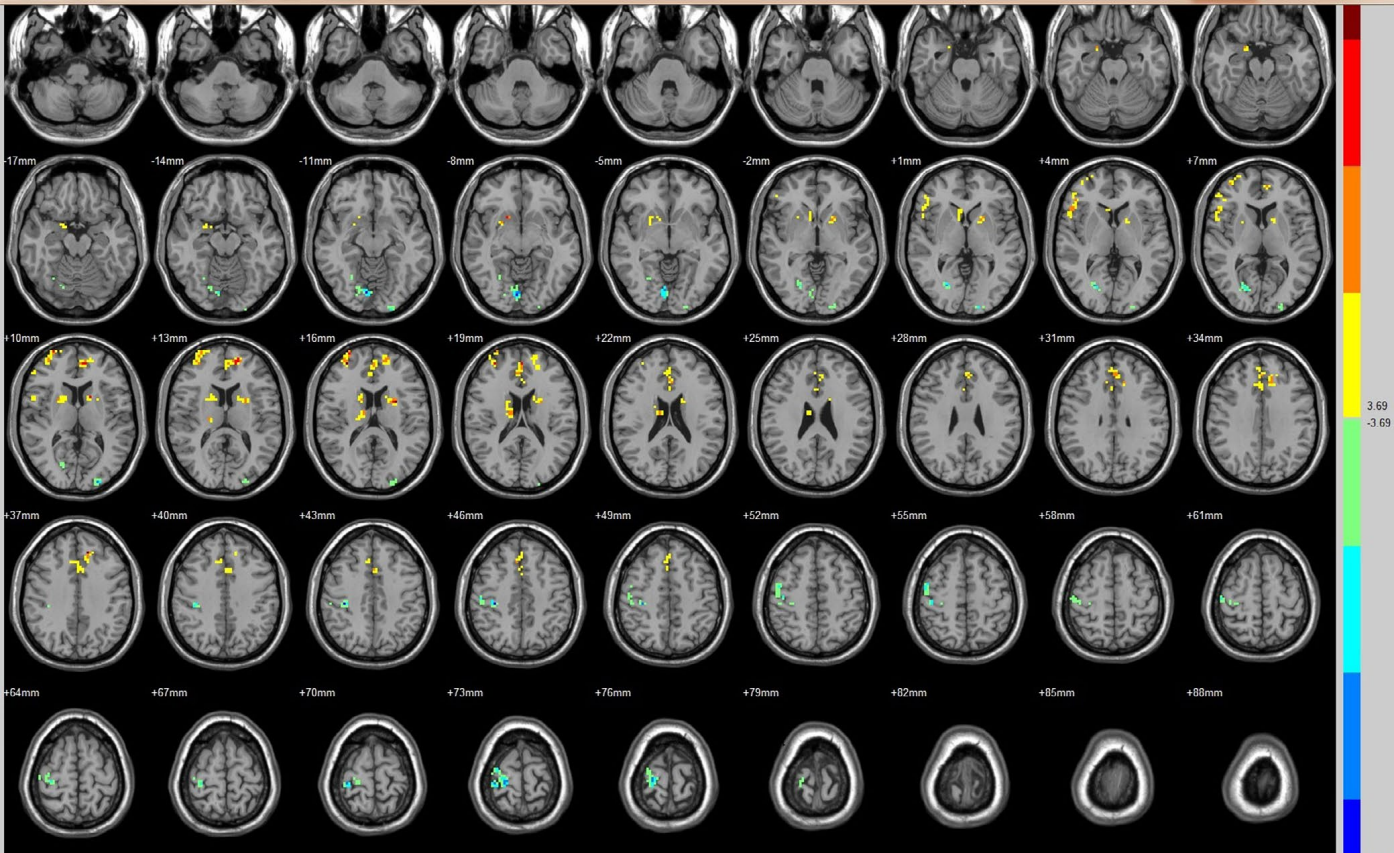
Brain areas exhibited significantly different FCs with the right MT + in PAs (postoperative vs. preoperative)

**FIGURE 7:**
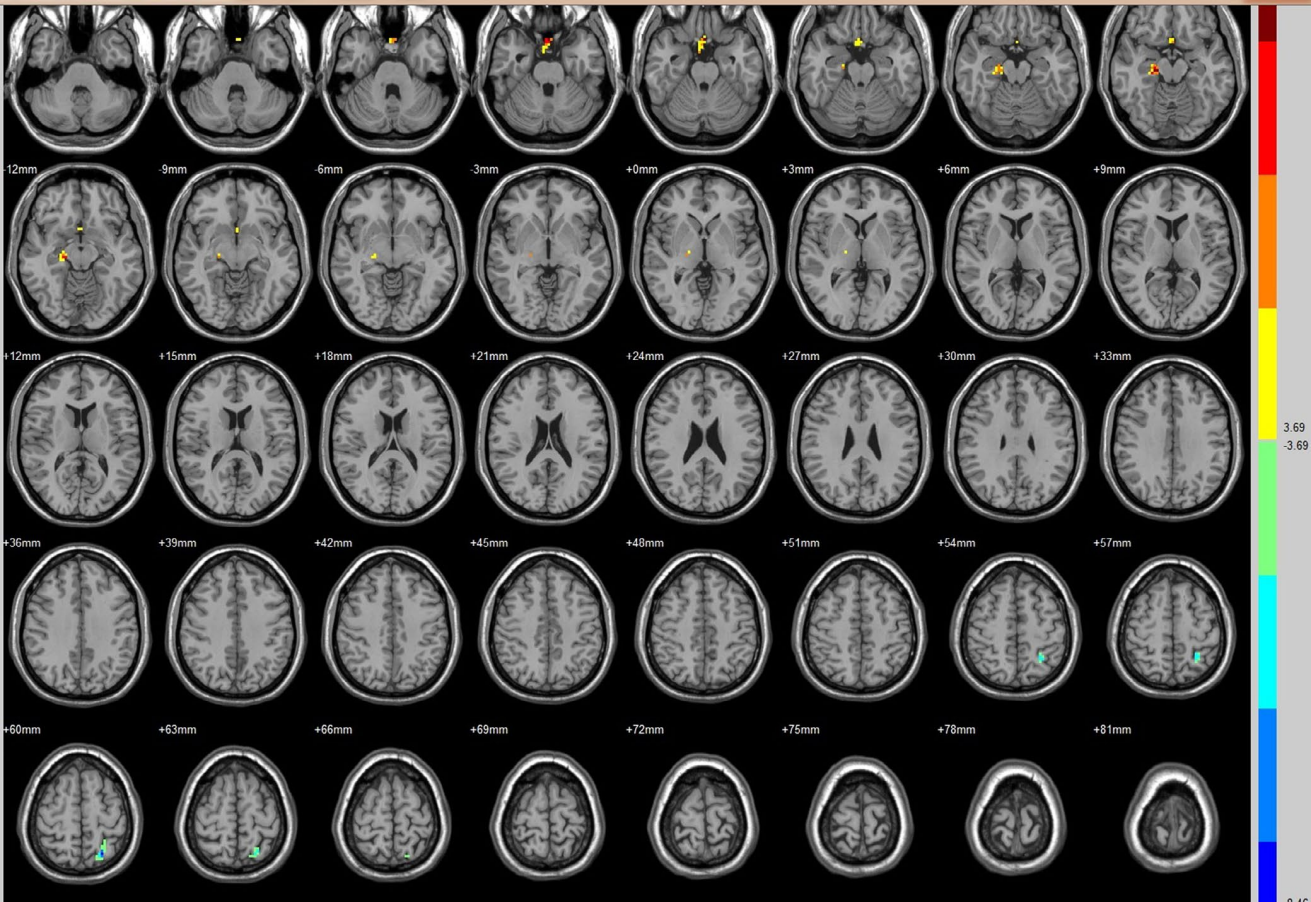
Brain areas exhibited significantly different FCs with the left FG in PAs (postoperative vs. preoperative)

**FIGURE 8:**
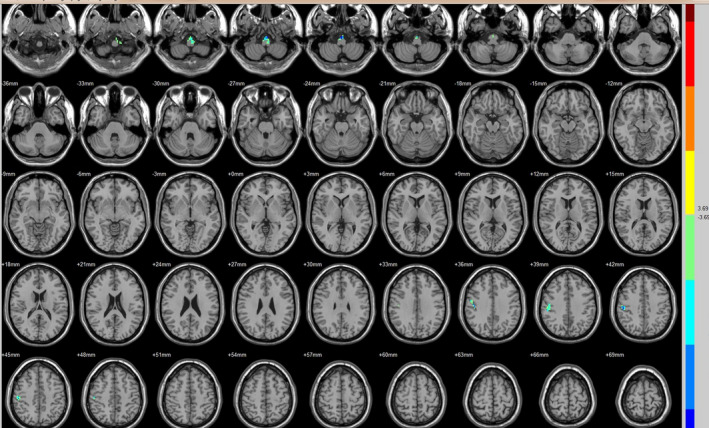
Brain areas exhibited significantly different FCs with the right FG in PAs (postoperative vs. preoperative)

Compared with the preoperative counterparts, decreased FC with left V1 was identified in the left paracentral lobule (Figure 1, Table 3). Decreased FC with right V1 was identified in the right lingual gyrus and left precentral gyrus (brodmann area (BA) 4) (Figure 2, Table 3). Decreased FC with left V2 was identified in the right superior temporal gyrus (BA 22) (Figure 3, Table 3). Decreased FC with right V2 was identified in the left fusiform gyrus, right middle occipital gyrus (BA 19), and left cuneus (Figure 4, Table 3). Decreased FC with left MT + was identified in the right inferior occipital gyrus, left lingual gyrus, and left superior frontal gyrus (Figure 5, Table 3). Decreased FC with right MT + was identified in the right lingual gyrus, right cuneus, left middle occipital gyrus, and right postcentral gyrus (Figure 6, Table 3). Decreased FC with left FG was identified in the left superior parietal lobule (BA 7) (Figure 7, Table 3). Decreased FC with right FG was identified in the medulla and right postcentral gyrus. (Figure 8, Table 3).

**TABLE 3:** Decreased FC after vision restoration

Seed	Brain region	Peak intensity	Peak MNI coordinate			Cluster size (voxels)
			*x*	*y*	*z*	
V1(L)	Paracentral Lobule(L)	−7.5049	−9	−21	45	61
V1(R)	Lingual Gyrus(R)	−6.8853	18	−66	−6	42
	Precentral Gyrus(L)/BA4	−6.6283	−27	−33	60	60
V2(L)	Superior Temporal Gyrus(R)/BA 22	−10.2326	66	−9	6	79
V2(R)	Fusiform Gyrus(L)	−5.4494	−21	−81	−6	40
	Middle Occipital Gyrus (R)/BA19	−8.0567	36	−93	9	50
	Cuneus(L)	−8.2541	−6	−90	15	128
MT(L)	Inferior Occipital Gyrus(R)	−8.3337	39	−87	−6	41
	Lingual Gyrus(R)	−8.8794	12	−63	−6	56
	Superior Frontal Gyrus(L)	−7.1932	0	−3	78	122
MT(R)	Lingual Gyrus(R)	−9.6612	9	−81	−12	52
	Cuneus(R)	−6.8596	24	−72	3	43
	Middle Occipital Gyrus(L)	−7.3186	−24	−96	9	41
	Postcentral Gyrus (R)	−8.7348	36	−27	42	177
FG(L)	Superior Parietal Lobule(L)/BA7	−7.4845	−24	−63	60	40
FG(R)	Medulla	−8.2256	0	−24	−51	50
	Postcentral Gyrus(R)	−7.4022	51	−24	42	45

#### 3.3.2 Increased FC in the patients after surgery

Compared with the preoperative counterparts, increased FC with right V1 was identified in the right middle frontal gyrus and left inferior parietal lobule (BA 40) (Figure 2, Table 4). Increased FC with left V2 was identified in the left declive, right lentiform nucleus, inferior frontal gyrus, and right middle frontal gyrus (Figure 3, Table 4). Increased FC with right V2 was identified in the right superior frontal gyrus (BA 11) and right cingulate gyrus (BA 32) (Figure 4, Table 4). Increased FC with left MT + was identified in the left cingulate gyrus (Figure 5, Table 4). Increased FC with right MT + was identified in the right putamen, right inferior frontal gyrus, right middle frontal gyrus, right thalamus, left medial frontal gyrus, left claustrum, and left superior frontal medial (Figure 6, Table 4). Increased FC with left FG was identified in the right rectal gyrus (BA 25) and right parahippocampal gyrus (Figure 7, Table 4).

**TABLE 4:** Increased FC after vision restoration

Seed	Brain region	Peak intensity	Peak MNI coordinate			Cluster size (voxels)
			*x*	*y*	*z*	
V1(R)	Middle Frontal Gyrus(R)	8.5609	48	33	27	57
	Inferior Parietal Lobule (L)/BA 40	7.1005	−45	−57	54	110
V2(L)	Declive(L)	7.3415	−33	−72	−24	85
	Lentiform Nucleus(R)	7.3157	18	9	3	56
	Inferior Frontal Gyrus(L)	9.4068	−36	12	15	40
	Middle Frontal(R)	8.5157	33	54	33	51
V2(R)	Superior Frontal Gyrus(R)/BA 11	9.2592	30	60	−12	49
	Cingulate Gyrus(R)/BA 32	7.976	15	3	45	44
MT(L)	Cingulate Gyrus(L)	6.136	0	−18	33	49
MT(R)	Putamen(R)	7.0856	18	12	−9	43
	Inferior Frontal Gyrus(R)	5.9378	54	21	6	55
	Middle Frontal Gyrus(R)	9.0008	33	51	15	71
	Thalamus(R)	6.4845	12	−9	18	62
	Medial Frontal Gyrus (L)	10.097	−12	36	36	154
	Claustrum(L)	8.7909	−27	3	15	54
	Superior Frontal Medial(L)	5.584	0	15	42	63
FG(L)	Rectal Gyrus(R)/BA 25	7.4383	0	15	−27	45
	Parahippocampal Gyrus(R)	6.9577	18	−21	−15	42

## 4 DISCUSSION

The V1 is a koniocortex (sensory‐type cortex) located in and around the calcarine fissure of the occipital lobe. Each hemisphere of the V1 receives information directly from its ipsilateral LGN and transmits information to the dorsal and ventral streams.

Our data show that the decreased FC with V1 and V2 was in the right lingual gyrus, left fusiform gyrus, right middle occipital gyrus (BA 19), left cuneus, right inferior occipital gyrus, and left lingual gyrus. The results imply neural disconnection within the visual cortex after improvement of vision in PAs. Decreased FC with MT + in the right inferior occipital gyrus, left lingual gyrus, right lingual gyrus, right cuneus, and left middle occipital gyrus implies neural disconnection between the occipital visual cortex and MT+. All these results may propose that the FC decreased in both the dorsal and ventral visual stream (V1, V2, MT+, FG, and other occipital gyrus) after visual restoration in PAs.

The dorsal stream originates from the V1 to V2 to MT+, and arrives at the inferior parietal lobule. This pathway is related with the detection of motion and location, and the control of the eyes and arms (Merigan & Maunsell, 1993; Tootell et al., 1998). Some studies have revealed abnormal function in vasomotor processing in patients without lesion in visual gyrus. The function of the dorsal visual pathway was abnormal in patients with amblyopia (Backus et al., 2001). The dorsal stream was impaired in patients with concomitant exotropia (Yan et al., 2010). Other studies have observed that both the ventral and dorsal visual pathway were disrupted in amblyopia subjects (Aaen‐Stockdale & Hess, 2008; Simmers et al., 2005). The dorsal visual pathways might be affected by visual restoration. The responses to motion in V5 bilaterally after visual restoration decreased after 3 days following surgery (Giulia et al., 2012). Consistent with the result, our study demonstrated decreased response within the dorsal stream after visual restoration in PAs. The ventral visual pathways might also be affected by visual restoration. The ReHo increased in part of the lateral occipital complex (LOC), lingual gyrus, and calcarine gyrus in patients with improved vision (Qian et al., 2015). The functional activity to faces in the right ventral visual pathway enhanced after visual restoration (Giulia et al., 2012). Contrary to the results, our data suggest that the FC was reduced within the ventral stream after visual restoration. In first study, the patients underwent the MRI scan at approximately 3 months after the operation. In our study, the MRI scan was performed 3 days after the operation, so the functional response of visual cortex may vary in different test time. In second study, only one patient was tested, and the task‐fMRI was used to evaluate the brain function. In our study, 14 patients were evaluated and RS‐fMRI was used, which may lead to different results. Our data show that the decreased FC involved in more regions of the dorsal stream than the ventral stream. The different response between the dorsal and ventral visual pathways may mean different mechanism developed by the visual restoration. The V1 and MT + could control the development of the dorsal and ventral pathway separately (Bourne, 2010). The development of the dorsal and ventral visual areas depended on different visual experiences (Qin et al., 2013). To the best of our knowledge, we do not find a possible reason for the decreased FC of the dorsal and ventral pathways affected by visual restoration. More fMRI studies are needed in the future.

The main visual information is relayed from the retina to the LGN to V1 (BA17) to V2 (BA18), and into higher‐order visual cortex. Some minimal visual information is transmitted from the retina to the pulvinar and LGN and directly to MT+/V5(Lyon et al., 2010; Warner et al., 2010), an alternative pathway that bypasses V1. Previous studies showed that MT + may be a potential substitute when a lesion in V1 occurs at early age (Bridge et al., 2008; Werth, 2006). The thalamus–MT bypass may play a compensatory role in the vision loss because of anterior vision pathway diseases (Mascioli et al., 2012). The retinothalamic pathways to MT + could play an important role in the drivers/modulators of visual perception in the adult (Laycock et al., 2007). Our data show that FC between thalamus and MT increased after improvement of vision, whereas no significant difference was identified between thalamus and V1. These results may indicate that the thalamus–MT/V5 bypass enhanced in the relay of visual information after visual restoration. MT may compensate following vision recovery at adulthood with intact V1.

Top‐down influences are transmitted across a series of descending pathways including the entire neocortex. The bottom‐up flow of visual information begin from V1 and ascend via two primary pathways, a ventral pathway and a dorsal pathway, and arrive at higher‐ order centers. The visual information is continually processed along this pathway. For every bottom‐up projections, there is a reciprocal top‐down projection that carries information about the behavioral context (Gilbert & Li, 2013; Xiong et al., 2016). These top‐down projections convey information about attention and expectation, modulate the lower‐level visual information, and manipulate perception of the visual background (Murphy et al., 2016). The role of these projections in cross‐modal activity in the visual cortex following visual restoration remains incompletely understood. Our results showed that the visual information in two‐stream pathway decreased after visual restoration. Compared with the preoperative counterparts, increased FC with V1, V2, and MT was identified in the right superior frontal gyrus(BA 11), the right middle frontal gyrus, left inferior frontal Lobe, and right inferior frontal gyrus. It was assumed the compensatory mechanism arised as feedback connection by top‐down influences.

Compared with the preoperative counterparts, increased FC with V1, V2, and MT was identified in the right putamen, right lentiform nucleus, right thalamus, and left claustrum. The right putamen, right lentiform nucleus, right thalamus, and left claustrum are subareas of the multisensory system (Cappe et al., 2009). The multisensory system at the cortical locations includes the parietal lobe, temporal lobe, frontal lobe, and insular. The multisensory system at the subcortical locations includes the superior colliculus and basal ganglia (globus pallidus, caudate nucleus, putamen nucleus, amygdaloid body, claustrum nucleus) (Brown et al., 1997; Cappe et al., 2009). The sensory‐specific thalamic structures play an important role in multisensory integration processes and behavior performances (Tyll et al., 2011). The putamen can integrate the neuronal interactions between visual recognition and articulatory areas (Seghier & Price, 2010). The claustrum nucleus connects with the visual cortex and integrates the information between the earlier visual cortex and vision‐related thalamic nucleus (Olson & Graybiel, 1980). The multisensory integration can modulate spatial attention and process the vision signal by top‐down influences (Convento et al., 2013; Wesslein et al., 2014). Our data show that the FC decreased in both dorsal and ventral pathways after vision recovery, so it is assumed that response of the multisensory region was enhanced as a type of feedback. The multisensory integration may play a role in the neural reconstruction for vision recovery.

Compared with the preoperative counterparts, decreased FC with left V1 was identified in the left paracentral lobule. Decreased FC with right V1 was identified in the left precentral gyrus (BA 4). Decreased FC with left V2 was identified in the right superior temporal gyrus (BA 22). Decreased FC with left MT was identified in the left superior frontal gyrus. Decreased FC with right MT was identified in the right postcentral gyrus. Decreased FC with left FG was identified in the left superior parietal lobule (BA 7). Decreased FC with right FG was identified in the medulla and right postcentral gyrus. Paracentral lobule, precentral gyrus (BA 4), postcentral gyrus, superior parietal lobule (BA 7), superior temporal gyrus (BA 22), superior frontal gyrus, MT, and FG are parts of a broader action observation network (AON) (Caspers et al., 2010). Observing others’ actions causes reaction in many sensorimotor cortexes that collectively consist in a network named the AON (Cross et al., 2009; Gazzola et al., 2007). The AON is proposed to contribute to the understanding of others’ actions by coping those actions into one's own motor system (Rizzolatti & Craighero, 2004). The AON may help implement the goal and intention understanding in others (Gazzola et al., 2006; Ortigue et al., 2010), which has been linked to social cognition (Cross et al., 2009; Kaplan & Iacoboni, 2006; Sobhani et al., 2012). The AON’s functional and structural connectivity at rest has been related to behavioral measures of social and motor skills (Fishman et al., 2015; Williams et al., 2017). Our results showed the decreased connection between the visual cortex and substrate of AON after vision restoration. The AON may be involved in the neural reconstruction for vision recovery.

In the FC analysis, we identify increased FC with ROIs in the bilateral cingulate gyrus (BA 32), and left medial frontal and superior frontal medial gyrus after visual restoration in PAs. All of these areas are subareas of the default mode network (DMN). The DMN plays a role in the detection and monitoring of both environmental events and internal mentation (Rudebeck et al., 2013) and mediates subject responsiveness and the saliency of external stimuli (Andrews‐Hanna et al., 2010; Leech & Sharp, 2014; Rudebeck et al., 2013; Wen et al., 2013). When the DMN detects the decreased visual cortical activity, the decreased deactivation in DMN may likely occur. The strong DMN activity is related with reduced visual cortical excitability (Mounder et al., 2013). Our results show that the FC decreased in visual cortex after vision recovery, so it may be justified to propose that decreased visual cortex activity in some way incur decreased DMN deactivation (stronger activity). However, the mechanism resulting in functional alteration in DMN after vision restoration is still to be elucidated.

Increased FC with FG in the right rectal gyrus (BA 25) and right parahippocampal gyrus mean increased neural connection after vision restoration. The rectal gyrus is subareas of the ventromedial prefrontal cortex (vmPFC). The vmPFC has been implicated in a variety of social, cognitive, and affective functions. The vmPFC modulates facial emotion recognition through its interactions with posterior cingulate cortex, precuneus, dorsomedial prefrontal cortex, and amygdala (Hiser & Koenigs, 2018). Our results propose that the increased response of rectal gyrus is related with the neurofunctional reorganization after the vision restoration. The parahippocampal gyrus is located in the inferior temporooccipital cortex, surrounding the hippocampus. The parahippocampal gyrus is involved in visual scenes (Mégevand et al., 2014), cognition (Aminoff et al., 2013), and spatial control (Aminoff et al., 2007). The parahippocampal gyrus has been suggested to control the processing of object and scene information (Staresina et al., 2011). A previous study showed the activation of the parahippocampal gyrus when a three‐dimensional spatial structure was presented (Henderson et al., 2008). Our results suggested that the vision restoration might enhance the function of the parahippocampal gyrus.

We also found an increase in the FC between the V2 and the cerebellum (declive). The cerebellum, which functionally interacts with the frontal eye fields (Kelly & Strick, 2003; Middleton & Strick, 2001), is also involved in the control of eye movements (Hayakawa et al., 2002; Nitta et al., 2008). Damage to the cerebellum can affect smooth pursuit eye movement (Straube et al., 1997). Our data proposed that the vision improvement leads to the increased function of the cerebellum.

However, there are several limitations in our study. First, the sample size is relatively small. Second, patients recruited had different time course and severity of the vision damage, which might affect the accuracy of the results. Third, the definition of MT + was based on previous studies instead of task‐fMRI definition, which may induce some bias. Fourth, there is no comparison in PA patients between 3 days and 3 or 6 months after treatment. In future studies, we will make well‐designed experiment to explore changes in brain function in PA patients with vision restoration.

## 5 CONCLUSIONS

Collectively, we showed rapid reorganization of neurofunctions in the vision‐related cortex of PA patients with visual improvement. Most subareas within the visual cortex exhibited decreased FC. The MT + exhibited enhanced FC with the thalamus, which may indicate an important role in the compensatory mechanism following visual improvement. The functional changes in subareas within DMN, AON and the multisensory system in PAs proposed that vision improvement may lead to function remodeling in higher‐order cortex beyond the visual cortex. However, more studies are needed to explore the mechanism of neural plasticity within the visual cortex, as well as the mechanism of the interaction between the visual and higher‐order cortex in patients with specific visual diseases.

## CONFLICTS OF INTEREST

The authors have no conflicts of interest to declare.

## AUTHOR CONTRIBUTIONS

Fuyu Wang: developed the main conceptual design of the study, experimental design, writing of the manuscript, edited manuscript. Jinli Jiang: supervised the data analyses and interpretation, edited manuscript, and final manuscript approval. Fuyu Wang, Peng Wang and Jinli Jiang: recruitment of patients, processed the fMRI data, performed the analysis, contributed to the manuscript, drafted the manuscript, analysis of the results, and to the writing of the manuscript. Yuyang Liu: manuscript editing, and contributed to the final version of the manuscript. Tao Zhou: compiled the references for the entire manuscript. Xianghui Meng: provided critical feedback on the results and conclusions and helped in editing the manuscript.

### Peer Review

The peer review history for this article is available at https://publons.com/publon/10.1002/brb3.1917.

## Data Availability

The data that support the findings of this study are available from the corresponding author upon reasonable request.
